# Duplicate gene expression in allopolyploid *Gossypium *reveals two temporally distinct phases of expression evolution

**DOI:** 10.1186/1741-7007-6-16

**Published:** 2008-04-16

**Authors:** Lex Flagel, Joshua Udall, Dan Nettleton, Jonathan Wendel

**Affiliations:** 1 Department of Ecology, Evolution, and Organismal Biology, Iowa State University, Ames, IA 50011, USA; 2 Department of Plant and Wildlife Sciences, Brigham Young University, Provo, UT 84602, USA; 3 Department of Statistics, Iowa State University, Ames, IA 50011, USA

## Abstract

**Background:**

Polyploidy has played a prominent role in shaping the genomic architecture of the angiosperms. Through allopolyploidization, several modern *Gossypium *(cotton) species contain two divergent, although largely redundant genomes. Owing to this redundancy, these genomes can play host to an array of evolutionary processes that act on duplicate genes.

**Results:**

We compared homoeolog (genes duplicated by polyploidy) contributions to the transcriptome of a natural allopolyploid and a synthetic interspecific F_1 _hybrid, both derived from a merger between diploid species from the *Gossypium *A-genome and D-genome groups. Relative levels of A- and D-genome contributions to the petal transcriptome were determined for 1,383 gene pairs. This comparison permitted partitioning of homoeolog expression biases into those arising from genomic merger and those resulting from polyploidy. Within allopolyploid *Gossypium*, approximately 24% of the genes with biased (unequal contributions from the two homoeologous copies) expression patterns are inferred to have arisen as a consequence of genomic merger, indicating that a substantial fraction of homoeolog expression biases occur instantaneously with hybridization. The remaining 76% of biased homoeologs reflect long-term evolutionary forces, such as duplicate gene neofunctionalization and subfunctionalization. Finally, we observed a greater number of genes biased toward the paternal D-genome and that expression biases have tended to increases during allopolyploid evolution.

**Conclusion:**

Our results indicate that allopolyploidization entails significant homoeolog expression modulation, both immediately as a consequence of genomic merger, and secondarily as a result of long-term evolutionary transformations in duplicate gene expression.

## Background

A hallmark of angiosperm genome organization is gene redundancy. Redundant genome segments have been identified in the composition and architecture of modern-day angiosperm genomes suggesting one or more ancient genome duplication events [1-3]. This has led to considerable interest in the evolution of the resulting duplicated genes. A key issue has been the identification of factors that enhance the retention of duplicate gene pairs and their potential for adaptive diversification or subfunctionalization (the partitioning of ancestral function). Mechanisms such as the maintenance of gene dosage and epistatic interactions [4,5] and epigenetically regulated expression subfunctionalization [6,7] have been implicated in aiding duplicate gene retention. These processes describe mechanisms of retention for *ancient *duplicate genes and naturally lead to questions about the evolutionary behavior of duplicate gene pairs in more recently formed polyploid species.

**Figure 1:**
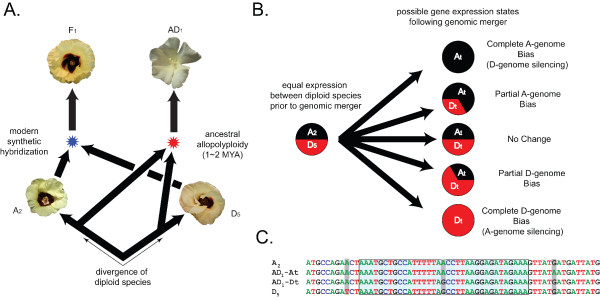
**Phylogenetic context and inference of homoeologous expression evolution in *Gossypium***. **(A) **Phylogentic relationships among the cotton accessions used in this study. An allopolyploidy event between A- and D-genome diploid species (red star) created modern allopolyploid *Gossypium hirsutum *(AD_1_). Using models of the ancenstral genome donors (A_2 _and D_5_), an interspecific diploid hybrid (F_1_) was created (blue star). Although not a perfect match, the model A- and D-genome donors are the best modern representatives of the diploids that underwent allopolyploidization to form AD_1 _and, as such, provide the best available reconstruction this ancient event. **(B) **Possible expression phenotypes and associated evolutionary inference. The far left pie represents equal expression among model diploid progenitor species (denoted by A_2 _and D_5_). Given this starting condition, several expression states are possible following allopolyploidy or hybridization. Some potential outcomes are indicated by the five pies on the right (A_t _and D_t _denote co-resident genomes, either in the hybrid or allopolyploid). **(C) **Detection of conserved homoeolog-specific single nucleotide polymorphism (SNPs). Given an alignment of expressed sequence tag (EST) sequences from orthologous genes from both diploid and allopolyploid genomes, species- and genome-specific SNPs (all SNPs highlighted in gray) can be detected. The middle SNP is an example of a genome-specific SNP. With this conserved SNP, homoeolog- and allele-specific microarray probes can be generated (potential microarray probe region highlighted in blue), and used to assay expression in allopolyploid and hybrid species.

Members of the cotton genus provide a phylogenetic framework to study the evolution of duplicate gene expression in recent polyploids because five diverse allopolyploid species are thought to have diverged from a single allopolyploidization event [8], and models of the ancestral diploid progenitor species (denoted by A_2 _and D_5_) have been identified (Figure 1A). In addition, extensive genomic resources, such as comprehensive expressed sequence tag (EST) libraries [9], microarray platforms [10,11], and BAC libraries [12] have greatly extended research capabilities. Synthesis of an F_1 _hybrid, combining the A- and D-genome diploid model species, offers the opportunity to untangle the effects of genomic merger from those arising from genome doubling and subsequent evolutionary change. This phylogenetic framework facilitates the study of gene expression from co-resident genomes on two temporal scales, from the onset of hybridization to a longer-term evolutionary timeframe encompassed by the natural allotetraploid species.

Adams et al [6] demonstrated that homoeolog expression in allotetraploid cotton has been strongly influenced by developmentally regulated, organ-specific silencing, resulting in subfunctionalization of the aggregate ancestral expression profile. This subfunctionalization may occur immediately after polyploidization or may arise over a longer period of evolutionary resolution [13,14]. The net effect is a process that appears to impose a form of selective retention on both homoeologs. Thus, expression subfunctionalization leads to prolonged duplicate gene retention, which may in turn enhance the potential for spatial, temporal, or functional divergence of duplicated genes.

Here we employ a novel microarray technology, which uses homoeolog specific probe sets, to assess the relative contribution of 1,383 homoeologous gene pairs to the transcriptome of natural allopolyploid *Gossypium hirsutum *and a synthetic, diploid F_1 _hybrid (denoted as AD_1 _and F_1_, respectively). We show that the two genomes contribute unequally to the total transcriptome of the allopolyploid. By comparing these entities we demonstrate that, for a substantial fraction of the genome, homoeolog expression biases occur immediately with the onset of genomic merger. In addition, a greater number of homoeolog expression biases appear in allopolyploid cotton that likely were not instigated by genomic merger. These findings indicate that upon allopolyploid formation, homoeolog expression biases happen in two, distinct temporal phases.

## Results

### Assessment of microarray quality

We analyzed the relative A- and D-genome contributions to the transcriptome of a synthetic F_1 _hybrid and AD_1 _allotetraploid cotton. This was done by comparing these mixed transcriptomes with the A_2 _and D_5 _model progenitors as well as with a 1:1 mix of A_2 _and D_5 _(Figure 1A). In total, 7,574 homoeolog-specific probe sets (around 33% of all possible) representing 1,383 unique EST contigs (hereafter referred to as genes) were identified as being reciprocally diagnostic with respect to identifying A- and D-genome specific expression in the F_1 _hybrid and allotetraploid cotton. Thus, using conservative measures (false discovery rate (FDR) ≤ 0.05), we recovered 1,383 diagnostic genes, representing 2.6% (see [15]) to 4% (J Hawkins, personal communication) of the genic content of the cotton genome. As expected, a principal component analysis on the natural log differences of A- and D-genome expression distinguished among all accessions, placing the AD_1_, F_1_, and 1:1 mix values intermediate between A_2 _and D_5 _along the first axis (see Figure S1 in Additional file 1). This indicates that the homoeolog-specific probes have performed as designed, and can be expected to yield useful estimates of A- and D-genome contributions to the transcriptome. Furthermore, quantitative mass-spectrometry validation of 12 homoeologous gene pairs from AD_1 _and 13 homoeologous gene pairs from F_1 _indicate that our findings regarding homoeolog-specific expression are reproducible (comparisons between platforms yielded *R*^2 ^values of 0.37 and 0.39 and *p*-values of 0.035 and 0.022, for AD_1 _and F_1_, respectively; see Figure S2 in Additional file 1).

### Detection of genome expression biases in polyploid and F_1 _*Gossypium*

**Figure 2:**
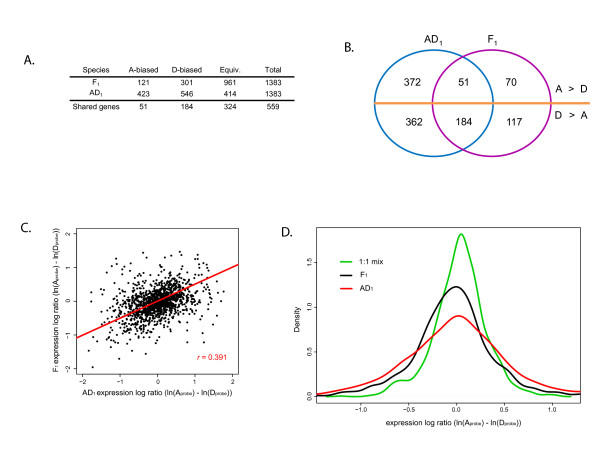
**Inferred contributions to the transcriptome by A- and D-genomes in a natural *Gossypium *allotetraploid and a synthetic diploid hybrid**. **(A) **A- and D-genome contribution to the transcriptome for 1,383 homoeologous/allelic gene pairs. Each gene pair categorized based on a linear model analysis of three replicate measures of genomic contribution. 'Shared genes' are those with expression patterns that are conserved between allotetraploid *G. hirsutum *(AD_1_) and the diploid F_1 _hybrid (F_1_). **(B) **Diagrammatic representation of the conservation of genes with biased expression. **(C) **Scatterplot comparing the homoeolog expression log ratios found in the natural allotetraploid AD_1 _to the synthetic F_1 _hybrid. Each point represents a single gene. The correlation (*r*) and best-fit line are indicated in red. This correlation has a *p*-value less than 2.2 × 10^-16^, indicating that it is significantly different from zero. **(D) **Kernel density estimates of the homoeolog expression log ratios for all 1,383 genes from the 1:1 mix (green line), F_1 _hybrid (black line), and AD_1 _allotetraploid (red line) cotton. This plot demonstrates an erosion of equal contribution from both genomes and a shift toward more extreme values in the allopolyploid when compared with the F_1 _or 1:1 mix.

For each gene, a linear model was fit to the three replicate measures of relative A- and D-genome contributions. Using FDR corrected *p*-values (FDR ≤ 0.15) from this model, each gene from the AD_1 _and F_1 _samples was categorized as 'A-biased' (log ratio ((ln(A_probe_) – ln(D_probe_)) statistically greater than 1:1 mix), 'D-biased' (log ratio statistically less than 1:1 mix) or 'Equivalent' (log ratio not statistically different from 1:1 mix); see Figure 2A. This categorization system is a rudimentary representation of the spectrum of homoeolog expression values, however, all categorizations presented here are based on known reference samples, which mitigates the effects of differential hybridization among homoeolog-specific probe pairs. In addition, this categorization is a *statistical *description of genome-specific transcript ratios and not a declaration of biological relevance (as pertaining to phenotype) of biases, which are unknown at present. Using this approach, many diagnostic gene pairs (29.9% (414 out of 1,383) of AD_1 _and 69.5% (961 out of 1,383) of F_1_) were inferred to be equivalently expressed in petals. We infer that these gene pairs showed no statistically significant change in homoeologous (or allelic for the F_1 _hybrid) contribution to the transcriptome relative to the *in vitro *mid-parent value. Among those genes exhibiting biased expression, there was an approximately 1.3*x *and 2.5*x *overrepresentation of the D-genome biased genes in petal tissues of AD_1 _and F_1_, respectively (Figure 2A, B). In addition, we detected 46 AD_1 _and 6 F_1 _genes that appear to be A-genome silenced and 69 AD_1 _and 5 F_1 _genes that are D-genome silenced, indicating a significant increase in silencing in the AD_1 _allopolyploid in both the A- and D-genomes. For a limited sampling of genes, expression biases comparable to those above have been demonstrated previously in cotton [6,13,14,16].

### Comparisons between hybridization (F_1_) and allopolyploidization (AD_1_)

The comparison between the artificially synthesized F_1 _hybrid and the 1–2 MY old natural allopolyploid, *G. hirsutum *(AD_1_), allows us to assess the role genomic merger plays in the allopolyploidization process [14,17]. The inclusion of model A- and D-genome diploid progenitors facilitates inference of ancestral expression states and, hence, the directionality and pace of expression evolution (Figure 1A, B). An additional temporal dimension to the analysis concerns homoeolog-specific expression biases detected in the AD_1 _allopolyploid that were also detected in the F_1 _hybrid (Figure 2B, C). This is demonstrated by both the sizable set of shared genes found within all expression categories (Figure 2A) and the positive correlation (Pearson's *r *= 0.391; *p*-value < 2.2 × 10^-16^) between estimates of genomic contribution in the F_1 _hybrid compared with those from the allopolyploid (Figure 2C). Overall around 24% (235 out of 969) of the genes with an A- or D-genome expression bias in the polyploid are also found to be biased in the same direction in the F_1 _hybrid. This indicates that a significant portion of the expression evolution associated with allopolyploidization may have accompanied the initial genomic merger.

An additional directional trend in the data is a tendency for the allopolyploid genes to exhibit more extreme expression biases (Figure 2D). Both the A- and D-genome biased genes demonstrate a greater number of more extremely biased AD_1 _genes, when compared with the F_1 _(1 and 18 gene(s), respectively, for shared A- and D-biased sets). In addition, paired *t*-test for equality between AD_1 _and F_1 _values confirm that the differences in means between AD_1 _and F_1 _are significantly different for D-biased genes (AD_1 _mean = -0.45 and F_1 _mean = -0.36; *p*-value = 6.63 × 10^-5^), and marginally non-significant for A-biased genes (AD_1 _mean = 0.46 and F_1 _mean = 0.37; *p*-value = 0.07). Thus, for genes with immediate expression biases toward one parental *Gossypium *genome, stabilization and evolution of the allopolyploid genome preferentially continues to enhance this initial bias.

## Discussion

### Genomic merger and duplicate gene expression evolution

It has long been thought that gene and genome duplication may serve as a key source of evolutionary innovation [18-23]. Recently, studies from a diverse array of organisms have demonstrated that gene duplication stimulates a variety of evolutionary outcomes [6,18,19,24-30]. These studies have demonstrated that following duplication, genes may evolve rapidly both at the sequence level and in their expression profile. It is thought that much of this change occurs as a result of the relaxation of purifying selection that occurs following duplication [18-20,22]. During this period of relaxed selection, duplicate genes either find new roles (*neofunctionalize*), partition ancestral roles (*subfunctionalize*) or accumulate deleterious mutations and decay as pseudogenes. These processes are thought to occur on an evolutionary timescale measured in thousands to millions of years; for example, it has been estimated that the average half-life of duplicate gene pairs is of the order of 3 to 7 MY for mammals, invertebrates, and plants [19]. Here we have demonstrated that expression divergence among many genes duplicated by allopolyploidy (AD_1_) is already apparent at the stage of interspecific genomic merger between two genomes (F_1_). These genes, with conserved homoeologous biases between an ancient allotetraploid and modern F_1 _hybrid, represent the proportion of loci we might expect to have immediately experienced expression alteration at the time of allopolyploid origin 1 to 2 MYA. These data indicate that the critical parameter 'time to subfunctionalization' [18,19], may actually be zero for a significant fraction of the genome in allopolyploid plants. Thus, we conclude that during allopolyploidization, genomic merger *per se *plays a crucial and persistent role in determining subsequent evolutionary trajectories in homoeolog expression patterns.

In addition to the foregoing set of genes inferred to have experienced instantaneous expression alteration as a consequence of genomic merger, an even larger class of genes did not exhibit shared expression biases in the F_1 _hybrid and AD_1 _allopolyploid. Specifically, 76% of the genes that displayed biased expression in AD_1 _were not biased in the intergenomic F_1_. Reciprocally, about 44% (187 out of 422) of the genes with biased expression in the F_1 _were not biased in AD_1_. These differences of expression bias may reflect (1) additional expression evolution in allopolyploid cotton since the interspecific genomic merger via the mechanisms of neo-, sub-, and non-functionalization [18,20], (2) differences between the parents of the F_1 _hybrid and the actual diploid progenitors of AD_1 _(Figure 1A); that is, the extant diploids are good models but they are not the actual progenitors of allopolyploid *Gossypium*, or (3) elimination of initial genome specific biases during chromosome doubling or subsequent evolution of the natural AD_1 _allopolyploid.

It has been shown that genes belonging to some functional categories are retained, following duplication, at a higher than expected rate [24]. As a corollary, it might be expected that gene function could also affect the likelihood of retention of expression bias. To explore this, we asked if genes from particular Gene Ontology (GO) [31] categories were over- or under-contributing to particular expression bias classes within the F_1 _hybrid and AD_1 _allopolyploid. Using the Blast2GO software [32], only two GO categories were found to be significantly over-represented and none were under-represented (FDR ≤ 0.05; data not shown). Both significant GO categories were inclusive high-level biological processes (cofactor metabolic process (GO:0051186); and coenzyme metabolic process (GO:0006732)), and were contained within the equivalently expressed genes from the F_1 _hybrid. We had, however, only limited power (that is, small numbers of genes within GO categories) to detect distortions between the observed and expected frequencies of GO categories. Thus, within our subset of analyzed genes, gene classification does not appear to be a strong predictor of the direction or degree of genome-specific bias, although the strength of this conclusion may be limited by our current sample size.

Taken together, these data indicate that a significant proportion (around 24%) of duplicate gene expression evolution, ascribed to allopolyploid cotton, could have been generated immediately during allopolyploid formation by genetic and epigenetic factors associated with interspecific genomic merger [4,5,33]. In addition, following allopolyploidy formation, subsequent duplicate gene evolution plays a large role in shaping homoeolog expression patterns. Thus, both immediate and long-term evolutionary processes contribute to homoeologous expression patterns. Based on this we speculate that expression-induced evolutionary novelty in allopolyploids occurs in two distinct modes: first, an immediate, massive, and saltational disruption of ancestral expression patterns accompanying the polyploidization process; and then a second, more gradual phase of expression evolution mediated by the mechanisms of duplicate gene evolution embodied in the traditional models [18,20] of the race between duplicate gene preservation and pseudogenization.

### 'Instantaneous subfunctionalization' could enhance duplicate gene retention

The signature of paleopolyploidy (ancient polyploidy) can be found in the genomes of all angiosperms [1-3,34-36]. In addition, a high proportion (30% to 50%) of paleologs (duplicate gene pairs arising from a paleopolyploidy event) can be retained for millions of years [3,18,34]. Adams and Wendel [14] have shown that A- and D-genome allelic pairs at the *Adh *locus display reciprocal silencing across multiple tissues in two *Gossypium *F_1 _hybrids. Thus, upon genomic merger ancestral gene expression domains are *immediately *partitioned and purifying selection is placed on both duplicate gene pairs, thereby increasing the probabilities of co-retention. To the extent that the results of Adams and Wendel [14] are mirrored by the present analysis, we have demonstrated that, in petals, around 17% (235 out of 1,383; Figure 2A) of the homoeologous gene pairs studied could potentially fit this model, by having been found to be biased immediately in the F_1 _and by having that bias retained throughout allopolyploidy. If we extrapolate this finding to the entire *Gossypium *genome, it would indicate that, following polyploidization, a large number of homoeologs could be retained by 'instantaneous subfunctionalization', occurring solely from the initial effects of genomic merger. Furthermore, given that these biases appear to have been maintained for about 1 to 2 MY following polyploidization, this immediate form of expression bias may play an underappreciated role in the retention of duplicate genes following whole genome duplication [6].

### Tissue-specific expression dominance

An intriguing aspect of the expression bias data is that for both natural AD_1 _allopolyploid and the interspecific F_1 _hybrid, a greater number of genes exhibited a D-genome bias than the reverse (Figure 2A, B). This bias favors the paternal D-genome genome, and stands in contrast to the recently reported A-genome bias described for ovular tissue [16]. To the best of the authors' knowledge, this is the most extensive example of widespread paternal dominance. When considered in light of the results of Yang et al [16], our data suggest that neither *Gossypium *genome is globally dominant with respect to expression, but that instead, each genome may have local dominance in certain tissue types or developmental stages. This finding confirms previous results in *Gossypium *[6,11] but differs from recent analysis of allotetraploid *Arabidopsis *[37]. In the latter study, leaf and flower bud tissues from a synthetic *Arabidopsis *allotetraploid were shown to exhibit dominance favoring only its *A. arenosa *parent, with genome-wide suppression of the *A. thaliana *parental contribution. In the tissues that have been studied in *Gossypium *and *Arabidopsis*, it appears that both species demonstrate biased parental contributions to the transcriptome, however, in *Gossypium *these biases can favor either parental genome, whereas in *Arabidoposis *only the *A. arenosa *parent has demonstrated dominance. These findings reflect the importance and perhaps *ad hoc *nature of specific genomic combinations and their interactions during allopolyploidization.

### Among instantaneously subfunctionalized genes, genomic biases tend to become more extreme during subsequent allopolyploid evolution

A notable observation in the present study is that genes showing biased expression patterns, tend to have more extreme biases in the AD_1 _allopolyploid (Figure 2D), including a much larger number of silenced genes (115 total), when compared with the F_1 _(11 total). One possible explanation for enhancement of genome-specific expression in allopolyploid cotton could be that immediately acting epigenetic effects become evolutionarily stabilized, either by natural selection or neutral processes. If this stabilization process is predisposed (through neutral or adaptive mechanisms) toward enhancing the initial expression bias, the result would be evolution toward a more extreme bias. This amplification of expression bias, which to our knowledge has not been described previously, may represent an additional factor underlying the genesis of phenotypic novelty in allopolyploid species.

## Conclusion

These results extend previous findings of homoeolog expression biases in hybrid and allotetraploid cotton [6,11,13,14,16]. By employing microarray technology to analyze a large number of genes, we describe the general phenomenon of genomic expression bias in both a modern synthetic F_1 _hybrid and an ancient allotetraploid. Furthermore, for petal tissues, these biases favor the parental D-genome and have become more extreme in the allotetraploid when compared with the F_1 _hybrid. By comparing homoeolog contributions to the transcriptome from the F_1 _hybrid and AD_1 _allotetraploid, it was possible determine the role of genomic merger in producing homoeolog expression biases. Given this comparison, we have shown that a significant fraction of the expression biases found in the allotetraploid is likely initiated immediately by genomic merger. A still larger fraction of the expression biases is inferred to have arisen from long-term evolutionary processes, thus implicating two temporally distinct phases of expression evolution following allopolyploidization.

## Methods

### Plant materials, experimental design, RNA isolation, and microarray preparation

Three replicate blocks of four *Gossypium *accessions (A_2 _| D_5 _| A_2_♀ X D_5_♂ F_1 _| AD_1_; Table 1) were grown in the Pohl Conservatory at Iowa State University, Ames, IA. These four accessions include representatives of both diploid progenitor genomes (A- and D-genomes) of natural allopolyploid cotton, their synthetic F_1 _hybrid, and an allotetraploid, respectively [8] (Figure 1A). Petals from all four accessions were harvested on the day of anthesis and three biological replicates were generated by pooling tissues from a minimum of eight flowers obtained from three individuals, or alternatively from a minimum of three flowers from a single individual if multiple individuals were not available (applicable only to F_1 _hybrid). RNA extractions were performed following a modified hot borate procedure optimized for *Gossypium *[38]. All RNA samples were quantified and visually assessed for degradation and DNA contamination via a Bioanalyzer (Agilent Technologies, Santa Clara, CA). From each pair of A_2 _and D_5 _replicates, an equimolar RNA mix (1:1 mix) was made. RNA samples were sent to NimbleGen Systems (Madison, WI), for cDNA synthesis, labeling, and hybridization to 15 microarrays, following proprietary protocols.

**Table 1:** Details of plant materials used in this study

Species name	Genome designation	Accession	Ploidy level	Location of origin
*G. arboreum*	A_2_	cv. AKA-8401	Diploid	Africa
*G. raimondii*	D_5_	Accession unnamed	Diploid	Peru
*G. arboreum *X *G. raimondii *F_1 _hybrid	A_2_♀ X D_5_♂	Accession unnamed	Diploid	Synthetic hybrid
*G. hirsutum*	AD_1_	cv. Maxxa	Allotetraploid	Mexico/Central America

Natural allotetraploid *Gossypium *evolved 1 to 2 MYA from diploid A- and D-genome progenitors, most similar to the modern species *G. arboreum *and *G. raimondii *[8, 43]. The A-genome parent is the inferred cytoplasmic donor to *G. hirsutum *[44, 45], and thus the F_1 _cross was created in the same manner, with A_2 _as the maternal parent.

### Homoeolog-specific microarray platform

We have designed and implemented a novel microarray platform capable of measuring homoeolog-specific expression in *Gossypium *species (Figure 1B). The utility of this design has been demonstrated with our first-generation arrays [11], but rapid developments in the depth of cotton EST resources, EST assembly quality, and microarray probe density enabled us to create a second-generation platform, which was used in this study. A description of the microarray design can be found in Additional file 1. This second-generation platform features oligonucleotide probe-pairs near 35 bases in length differing by an A- or D-genome homoeolog-specific single nucleotide polymorphism (SNP) at their middle base (Figure 1C, box). Thus, the microarray platform has the ability to measure expression levels separately for each homoeolog, detected by the corresponding homoeolog-specific probe.

### Statistical analysis

Raw data values for each microarray were natural log transformed, median centered, and scale normalized across all arrays prior to analysis. For each homoeolog probe pair the difference of natural logs of the A- and D-homoeolog-specific probe was calculated ((ln(A_probe_) – ln(D_probe_); hereafter referred to as log ratio). Using this approach, positive values indicate an A-genome bias, whereas negative values indicate a D-genome bias. A linear model including effects for replication and genotype was fit to the log ratio data from each probe to identify the subset of probes that diagnostically detected homoeolog-specific expression. This was done by filtering for only those probes in which the log ratio for A_2 _was significantly (FDR ≤ 0.05; see [39]) and appreciably greater (fold change of at least 1.5) than the 1:1 mix of A_2 _and D_5_, and the 1:1 mix log ratio was significantly and appreciably greater than D_5 _(A_2 _> 1:1 mix > D_5_). The resulting, empirically identified, probe sets can diagnose homoeolog-specific expression levels within transcriptionally mixed A- and D-genome hybrid and allopolyploid RNA samples.

Following the identification of all diagnostic probes, contig-level log ratio values were determined by calculating a robust average of the log ratio values from all diagnostic probe sets within a contig using Tukey's Biweight method. A linear model including effects for replication and genotype was fit to this contig-level data, allowing the estimation of all possible contrasts between A_2_, D_5_, 1:1 mix, AD_1_, and the F_1 _hybrid. The contrasts between the AD_1 _and F_1 _samples and the 1:1 mix allow us to diagnose change relative to the *in vitro *mid-parent value of the A_2 _and D_5 _diploids. In addition, these contrasts account for the specific hybridization kinetics of each probe, when faced with a genomically mixed transcript pool. This is useful, as it can factor out non-linear competitive interactions that may occur as a result of the interaction between A- and D-genome transcripts.

Given the distributions of *p*-values from AD_1 _versus 1:1 mix and F_1 _versus 1:1 mix contrasts, we estimated the expected number of true null hypotheses, using the procedure of Nettleton et al [40]. It was determined that approximately 495 and 884 genes were true nulls, and thus not statistically different in mean log ratio from the 1:1 mix, for AD_1 _and F_1_, respectively. Using these estimates from the AD_1 _versus 1:1 mix and F_1 _versus 1:1 mix contrasts, we selected an FDR threshold for significance [41] of 0.15 to strike a reasonable balance between the expected number of false positives and false negatives. FDR significance thresholds of 0.05 and 0.10 were examined as well and can be found in Table S1A, B in Additional file 1.

Using the A_2 _and D_5 _diploids as a reference measure of pure A- or D-genome expression gives us the ability to discover cases of genome-specific gene silencing in both AD_1 _and F_1_. These putative cases of silencing can be detected as log ratio values that are greater than or equal to the A_2 _diploid parent or less than or equal to the D_5 _diploid parent. Using this definition of silencing, we were able to detect gene silencing in both the AD_1 _and F_1 _accessions.

### Validation of microarray results with Sequenom quantitative mass-spectrometry

Validation of our microarray results was performed for 13 randomly selected homoeologous gene pairs using Sequenom quantitative mass-spectrometry following the methods of Stupar and Springer [42]. Aliquots of RNA transcripts used for microarray hybridizations were analyzed for A- and D-genome contributions to the transcriptome for AD_1 _and F_1 _samples (the validation design can be found in Additional file 1). Briefly, the Sequenom technology amplifies A- and D-derived cDNA transcripts in parallel, and then quantifies relative homoeolog abundance based on matrix-assisted laser desorption/ionization time-of-flight mass-spectrometry. All Sequenom assays were conducted at the University of Minnesota Genotyping Facility.

## Authors' contributions

LF, JU, and JW designed the research. JU and JW designed the microarray platform. LF and JU performed the research. LF and DN analyzed the data. LF and JW drafted the manuscript. All authors participated in editing the manuscript and approved the final version.

## Supplementary Material

Additional file 1 This file includes additional information about microarray construction and the design of the Sequenom validation experiment. In addition, there are two tables (Table S1A and B) detailing the results of alternative *q*-value thresholds and two figures (Figures S1 and S2), including a principal component analysis of all expression data and the results of the Sequenom mass spectrometry validation experiment. Click here for file

## References

[B1] Bowers JE, Chapman BA, Rong J, Paterson AH (2003). Unravelling angiosperm genome evolution by phylogenetic analysis of chromosomal duplication events. Nature.

[B2] Lockton S, Gaut BS (2005). Plant conserved non-coding sequences and paralogue evolution. Trends Genet.

[B3] Wendel JF (2000). Genome evolution in polyploids. Plant Mol Biol.

[B4] Birchler JA, Riddle NC, Auger DL, Veitia RA (2005). Dosage balance in gene regulation: biological implications. Trends Genet.

[B5] Veitia RA (2005). Paralogs in polyploids: one for all and all for one?. Plant Cell.

[B6] Adams KL, Cronn R, Percifield R, Wendel JF (2003). Genes duplicated by polyploidy show unequal contributions to the transcriptome and organ-specific reciprocal silencing. Proc Natl Acad Sci USA.

[B7] Rodin SN, Riggs AD (2003). Epigenetic silencing may aid evolution by gene duplication. J Mol Evol.

[B8] Wendel JF, Cronn R (2003). Polyploidy and the evolutionary history of cotton. Adv Agron.

[B9] Udall JA, Swanson JM, Haller K, Rapp RA, Sparks ME, Hatfield J, Yu Y, Wu Y, Dowd C, Arpat AB, Sickler BA, Wilkins TA, Guo JY, Chen XY, Scheffler J, Taliercio E, Turley R, McFadden H, Payton P, Klueva N, Allen R, Zhang D, Haigler C, Wilkerson C, Suo J, Schulze SR, Pierce ML, Essenberg M, Kim H, Llewellyn DJ, Dennis ES, Kudrna D, Wing R, Paterson AH, Soderlund C, Wendel JF (2006). A global assembly of cotton ESTs. Genome Res.

[B10] Udall JA, Flagel LE, Cheung F, Woodward AW, Hovav R, Rapp RA, Swanson JM, Lee JJ, Gingle AR, Nettleton D, Town CD, Chen ZJ, Wendel JF (2007). Spotted cotton oligonucleotide microarrays for gene expression analysis. BMC Genomics.

[B11] Udall JA, Swanson JM, Nettleton D, Percifield RJ, Wendel JF (2006). A novel approach for characterizing expression levels of genes duplicated by polyploidy. Genetics.

[B12] Comparative Evolutionary Genomics of Cotton. http://cottonevolution.info.

[B13] Adams KL, Percifield R, Wendel JF (2004). Organ-specific silencing of duplicated genes in a newly synthesized cotton allotetraploid. Genetics.

[B14] Adams KL, Wendel JF (2005). Allele-specific, bidirectional silencing of an alcohol dehydrogenase gene in different organs of interspecific diploid cotton hybrids. Genetics.

[B15] Rabinowicz PD, Citek R, Budiman MA, Nunberg A, Bedell JA, Lakey N, O'Shaughnessy AL, Nascimento LU, McCombie WR, Martienssen RA (2005). Differential methylation of genes and repeats in land plants. Genome Res.

[B16] Yang S, Cheung F, Lee JJ, Ha M, Wei NE, Sze S-H, Stelly DM, Thaxton P, Triplett B, Town CD, Chen ZJ (2006). Accumulation of genome-specific transcripts, transcription factors and phytohormonal regulators during early stages of fiber cell development in allotetraploid cotton. Plant J.

[B17] Hegarty MJ, Barker GL, Wilson ID, Abbott RJ, Edwards KJ, Hiscock SJ (2006). Transcriptome shock after interspecific hybridization in *Senecio *is ameliorated by genome duplication. Curr Biol.

[B18] Force A, Lynch M, Pickett FB, Amores A, Yan Y-l, Postlethwait J (1999). Preservation of duplicate genes by complementary, degenerative mutations. Genetics.

[B19] Lynch M, Conery JS (2000). The evolutionary fate and consequences of duplicate genes. Science.

[B20] Ohno S (1970). Evolution by Gene Duplication.

[B21] Ohta T (1987). Simulating evolution by gene duplication. Genetics.

[B22] Stephens SG (1951). Possible significances of duplication in evolution. Adv Genet.

[B23] Walsh JB (1995). How often do duplicated genes evolve new functions?. Genetics.

[B24] Blanc G, Wolfe KH (2004). Functional divergence of duplicated genes formed by polyploidy during *Arabidopsis *evolution. Plant Cell.

[B25] Casneuf T, De Bodt S, Raes J, Maere S, Van de Peer Y (2006). Nonrandom divergence of gene expression following gene and genome duplications in the flowering plant *Arabidopsis thaliana*. Genome Biol.

[B26] Duarte JM, Cui L, Wall PK, Zhang Q, Zhang X, Leebens-Mack J, Ma H, Altman N, dePamphilis CW (2006). Expression pattern shifts following duplication indicative of subfunctionalization and neofunctionalization in regulatory genes of Arabidopsis. Mol Biol Evol.

[B27] Gu X, Zhang Z, Huang W (2005). Rapid evolution of expression and regulatory divergences after yeast gene duplication. Proc Natl Acad Sci USA.

[B28] Gu Z, Rifkin SA, White KP, Li W-H (2004). Duplicate genes increase gene expression diversity within and between species. Nat Genet.

[B29] Hughes MK, Hughes AL (1993). Evolution of duplicate genes in a tetraploid animal, *Xenopus laevis*. Mol Biol Evol.

[B30] Kellis M, Birren BW, Lander ES (2004). Proof and evolutionary analysis of ancient genome duplication in the yeast *Saccharomyces cerevisiae*. Nature.

[B31] Ashburner M, Ball CA, Blake JA, Botstein D, Butler H, Cherry JM, Davis AP, Dolinski K, Dwight SS, Eppig JT, Harris MA, Hill DP, Issel-Tarver L, Kasarskis A, Lewis S, Matese JC, Richardson JE, Ringwald M, Rubin GM, Sherlock G (2000). Gene Ontology: tool for the unification of biology. Nat Genet.

[B32] Conesa A, Gotz S, Garcia-Gomez JM, Terol J, Talon M, Robles M (2005). Blast2GO: a universal tool for annotation, visualization and analysis in functional genomics research. Bioinformatics.

[B33] Chen ZJ, Ni Z (2006). Mechanisms of genomic rearrangements and gene expression changes in plant polyploids. Bioessays.

[B34] Blanc G, Wolfe KH (2004). Widespread paleopolyploidy in model plant species inferred from age distributions of duplicate genes. Plant Cell.

[B35] Cui L, Wall PK, Leebens-Mack JH, Lindsay BG, Soltis DE, Doyle JJ, Soltis PS, Carlson JE, Arumuganathan K, Barakat A, Albert VA, Ma H, dePamphilis CW (2006). Widespread genome duplications throughout the history of flowering plants. Genome Res.

[B36] Vision TJ, Brown DG, Tanksley SD (2000). The origins of genomic duplications in *Arabidopsis*. Science.

[B37] Wang J, Tian L, Lee H-S, Wei NE, Jiang H, Watson B, Madlung A, Osborn TC, Doerge RW, Comai L, Chen ZJ (2006). Genomewide nonadditive gene regulation in *Arabidopsis *allotetraploids. Genetics.

[B38] Wan CY, Wilkins TA (1994). A modified hot borate method significantly enhances the yield of high-quality RNA from cotton (*Gossypium hirsutum *L.). Anal Biochem.

[B39] Benjamini Y, Hochberg Y (1995). Controlling the false discovery rate: a practical and powerful approach to multiple testing. J Roy Statist Soc Ser B Stat Methodol.

[B40] Nettleton D, Hwang JTG, Caldo RA, Wise RP (2006). Estimating the number of true null hypotheses from a histogram of p values. J Agric Biol Environ Stat.

[B41] Storey JD, Tibshirani R (2003). Statistical significance for genomewide studies. Proc Natl Acad Sci USA.

[B42] Stupar RM, Springer NM (2006). Cis-transcriptional variation in maize inbred lines B73 and Mo17 lead to additive expression patterns in the F1 hybrid. Genetics.

[B43] Senchina DS, Alvarez I, Cronn RC, Liu B, Rong J, Noyes RD, Paterson AH, Wing RA, Wilkins TA, Wendel JF (2003). Rate variation among nuclear genes and the age of polyploidy in *Gossypium*. Mol Biol Evol.

[B44] Wendel JF (1989). New World tetraploid cottons contain Old World cytoplasm. Proc Natl Acad Sci USA.

[B45] Wendel JF, Albert VA (1992). Phylogenetics of the cotton genus (*Gossypium*): character-state weighted parsimony analysis of chloroplast-DNA restriction site data and its systematic and biogeographic implications. Syst Bot.

