# Supplementary material for: Duplicate gene expression in allopolyploid Gossypium reveals two temporally distinct phases of expression evolution
Source: BMC Biol. 2008 Apr 16;6:16. doi: 10.1186/1741-7007-6-16 (PMC2330141; doi:10.1186/1741-7007-6-16)

Supplemental Material

SI Methods:

Microarray Design:

Homoeolog-specific probes were created by first assembling EST contigs from A_2, D_5, and AD_1 (Table 1) libraries and then identifying homoeolog-specific SNPs within these contigs. These SNPs represent nucleotide differences between the A and D genome orthologs, and offer the possibility of diagnosing the genomic origin of transcripts in the allopolyploid nucleus. Additionally, when available, AD_1 EST sequences confirmed the conservation of A- and D-genome-specific SNPs in the allopolyploid species. Using this strategy, 11,399 high-quality SNPs were identified, encompassing 2029 contigs. For each of these 11,399 SNPs, complimentary plus and minus strand A and D homoeolog-specific probes sets were designed, generating in total 22,798 probes sets, and 45,596 unique probes.

Mass-Spectrometry Validation Experimental Design and Methodology:

Cotton petal RNA samples were converted to cDNA and PCR amplified with multiplex primer sets, which targeted 13 genes from the homoeolog-specific microarray results. Each biological replicate was split into three technical replicates resulting in 9 total replicate measures for each species (3 bio. reps. X 3 tech. reps.). Amplified multiplex products were sent to the University of Minnesota for homoeolog-specific MALDI-TOF mass-spectrometry quantification using a Sequenom (San Diego, CA) MassARRAY device. The mean value for each of the nine replicates was determined and compared to the estimates derived from the homoeolog-specific microarray (Supp. Fig. 3)

Supp. Table 1. A- and D-genome contribution to the transcriptome at FDR thresholds of 0.05 (*A*) and 0.1 (*B*). Each gene pair categorized based on a linear model analysis of three replicate measures of genomic contribution. "Shared genes" are those with expression patterns that are conserved between *G. hirsutum* and the diploid hybrid.

Supp. Figure 1. Principle Component Analysis of natural log differences between A- and D-genome specific probe expression levels. All three replicate samples of each genotype are represented. Character symbols for the five genomic samples are as follows: "A" = A_2, "D" = D_5, "F" = F_1 hybrid, "M" = 1:1 A_2:D_5 RNA mix, and "P" = AD_1 allotetraploid. The proportion of the total variance explained by each principle component is listed on the corresponding axis.

Supp. Figure 2. Validation of homoeolog expression results for AD_1 and F_1 accessions. (*A*) A comparison of results for 13 randomly chosen genes. All NimbleGen (microarray) values are expressed as the log ratio ($\ln(A_{probe}) - \ln(D_{probe})$), whereas the Sequenom (mass-spectrometry) values are expressed as the proportion of the transcriptome contributed by the A-genome. Thus both metrics result in analogous interpretations of the different data types (ie. for both technologies, larger values reflect greater A-genome contribution to the transcriptome, and smaller values reflect greater D-genome contribution). Scatter plots of validation results for AD_1 and F_1 (*B*) with their associated best-fit line, R^2 value, and *p*-value.

Supp. Table 1A: FDR threshold = 0.05

	A-bias	D-bias	Equiv.	Total
F_1	37	76	1270	1383
AD_1	283	380	720	1383
shared	13	47	683	743

Supp. Table 1B: FDR threshold = 0.1

	A-bias	D-bias	Equiv.	Total
F_1	76	186	1121	1383
AD_1	358	472	553	1383
shared	33	112	483	628

Supp. Figure 1:

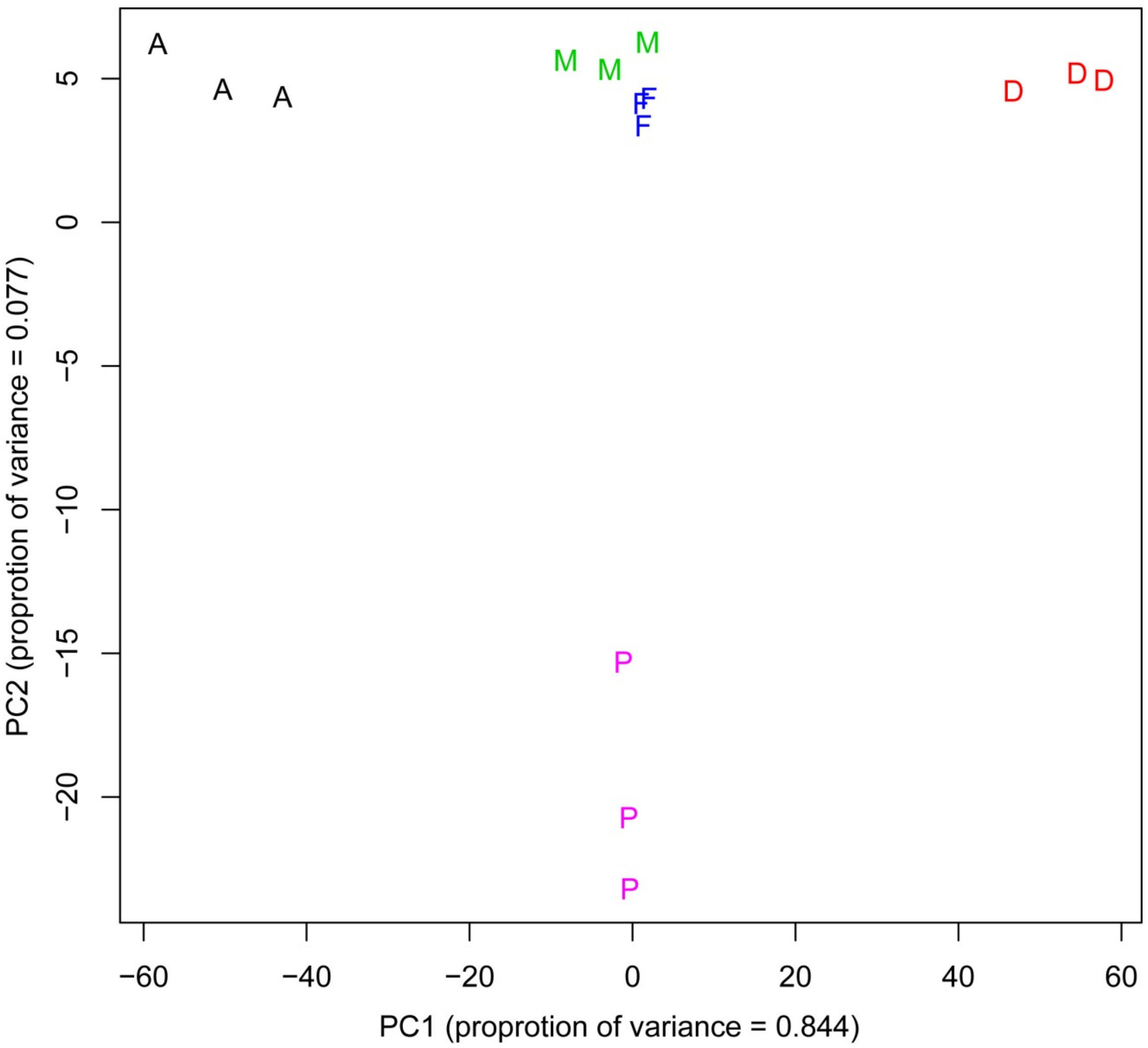

Supp. Figure 2A:

contig	SNP position	AD1 Sequenom % A	AD1 NimbleGen ln(a) - ln(d)	F1 Sequenom % A	F1 NimbleGen ln(a) - ln(d)
COTTON16_00001_062	1928	0.543	0.728	0.549	0.551
COTTON16_00024_03	2070	0.293	-0.867	0.097	-0.766
COTTON16_00076_06	860	NA	NA	0.449	-0.448
COTTON16_00174_02	802	0.734	0.562	0.408	-0.251
COTTON16_00285_02	685	0.425	0.09	0.395	0.343
COTTON16_00690_02	916	0.469	0.725	0.384	-0.946
COTTON16_01391_01	705	0.607	0.01	0.554	0.059
COTTON16_07872_01	1017	0.311	-0.632	0.255	-0.405
COTTON16_07872_01	1185	0.313	-0.66	0.21	-0.433
COTTON16_09095_01	1544	0.504	0.59	0.586	0.691
COTTON16_21601_01	747	0.557	0.026	0.515	0.04
COTTON16_25466_01	1125	0.482	0.425	0.531	-0.412
COTTON16_32946_01	1145	0.702	-0.067	0.564	-0.107

Supp. Figure 2B:

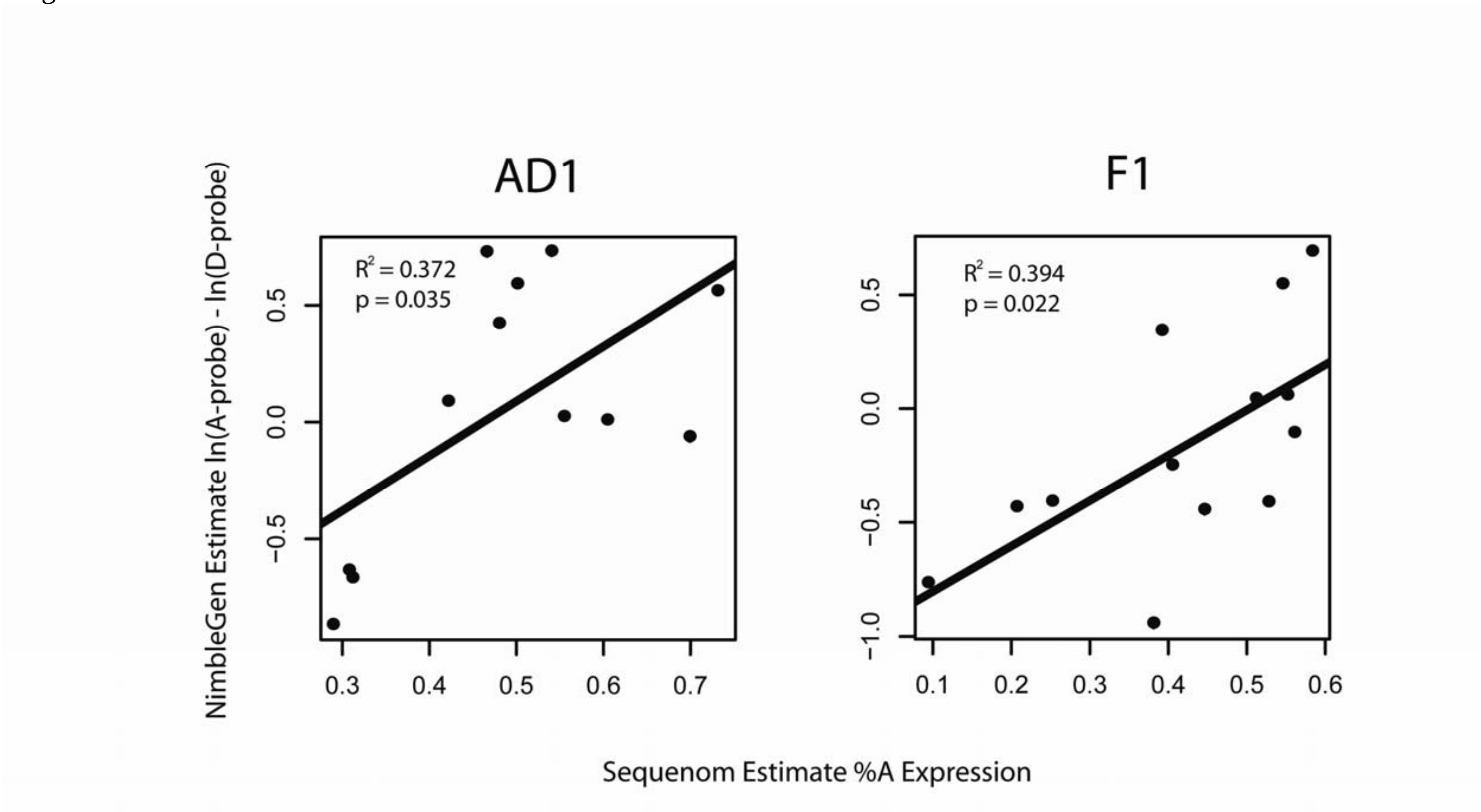

Supplement: Additional file 1 — This file includes additional information about microarray construction and the design of the Sequenom validation experiment. In addition, there are two tables (Table S1A and B) detailing the results of alternative q-value thresholds and two figures (Figures S1 and S2), including a principal component analysis of all expression data and the results of the Sequenom mass spectrometry validation experiment. [file 1741-7007-6-16-S1.pdf]
